# Epitaxial Growth of Surface Perforations on Parallel Cylinders in Terraced Films of Block Copolymer/Homopolymer Blends

**DOI:** 10.1021/acs.langmuir.4c00385

**Published:** 2024-03-29

**Authors:** Ya-Sen Sun, Yi-Qing Jian, Shin-Tung Yang, Hsiao-Fang Wang, Belda Amelia Junisu, Chun-Yu Chen, Jhih-Min Lin

**Affiliations:** † Department of Chemical Engineering, National Cheng Kung University, Tainan 701, Taiwan; ‡ Department of Chemical and Materials Engineering, National Central University, Taoyuan 32001, Taiwan; § National Synchrotron Radiation Research Center, Hsinchu 30076, Taiwan

## Abstract

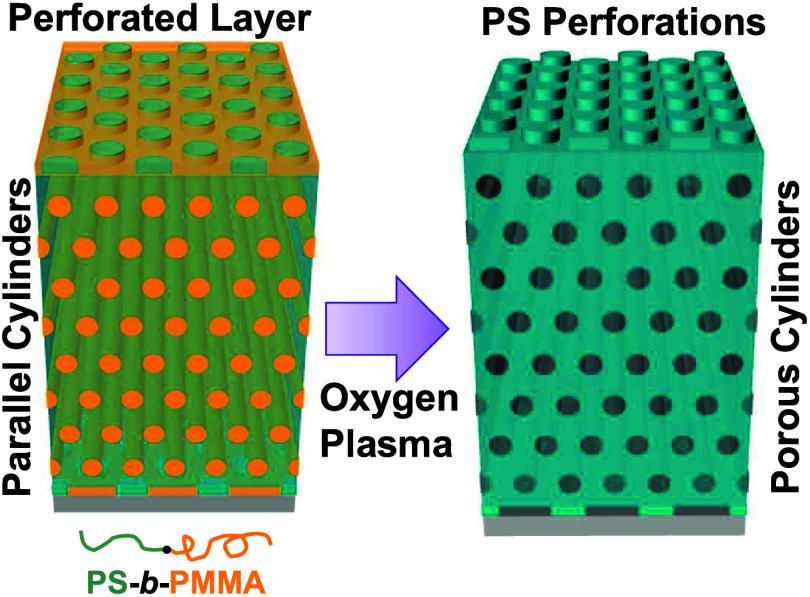

Due to incommensurability between initial thickness and interdomain distance, thermal annealing inevitably produces relief surface terraces (islands and holes) of various morphologies in thin films of block copolymers. We have demonstrated three kinds of surface terraces in blend films: polygrain terraces with diffuse edges, polygrain terraces with step edges, and pseudo-monograin terraces with island coarsening. The three morphologies were obtained by three different thermal histories, respectively. The thermal histories were imposed on blend films, which were prepared by mixing a homopolystyrene (hPS, 6.1 kg/mol) with a weakly segregated, symmetry polystyrene-block poly(methyl methacrylate) (PS-*b*-PMMA, 42 kg/mol) followed by spin coating. At a given weight-fraction ratio of PS-*b*-PMMA/hPS = 75/25, the interior of the blend films forms parallel cylinders. Nevertheless, the surface of the blend films is always dominated by a skin layer of perforations, which epitaxially grow on top of parallel cylinders. By oxygen plasma etching at various time intervals to probe interior nanodomains, the epitaxial relationship between surface perforations and parallel cylinders has been identified by a scanning electron microscope.

## Introduction

Thin block copolymer (BCP) films have received significant attention because surface fields and spatial confinement result in morphological diversity, dimensional tunability, and nanodomain orientation.^1−35^ For BCPs in bulk, the phase behavior is mainly determined by the segregation strength and volume fraction of constituent blocks.^36^ In contrast, several nonbulk morphologies have been found in thin films of lamellae- and cylinder-forming BCP bulks when those films are supported on a solid with strong surface fields.^2−7^ Thus, surface fields on the free surface and substrate interface have been considered to interpret how the nonbulk morphologies are finely tailored.

Lamellae and cylinders have been frequently studied to understand how surface fields and spatial confinement influence domain orientations and morphologies in thin films.^37^ If both the free surface and substrate interface are neutral, perpendicular orientation is preferential. Because of perpendicular orientation, there is no film incommensurability between initial thickness and interdomain distance. However, if one surface is neutral and the other is selective, perpendicular, parallel, or mixed, then orientation is favored. These different orientations depend on an interplay between neutral and selective surfaces. If films are confined on two attractive surfaces, parallel orientation dominates over perpendicular orientation for nanodomains in films.

For parallel or mixed orientation, films tend to form relief terraces (holes and islands) if their initial thickness is incommensurate with the interdomain distance of parallel nanodomains.^38−41^ For terraced films, the morphologies are more complicated. Multiple phases may coexist vertically or laterally in some terraced films. The multiple phases can be stabilized by the strength of either the free surface or the substrate interface and by the film thickness. Knoll et al. have found wetting layer, perforated lamella, parallel cylinders with necks, and lamella in films of cylinder-forming polystyrene-*block*-polybutadiene-*block*-polystyrene triblock copolymers.^2,4^ Some nonbulk structures can coexist vertically and laterally in the same film of terraced thickness. They also demonstrated that the cylindrical shape is flexible and adjustable under various surface fields and spatial confinement. Thus, in addition to perforated layers, cylinders with necks and cylinders with modulated shapes can also be obtained as thin films.

In experiments investigating morphologies in thin films of cylinder-forming BCPs, relief terraces frequently form on a solid substrate with attractive interactions with either constituent block. Relief terraces are due to film incommensurability between initial thickness and interdomain distance. In that case, the edge of relief terraces reveals a different morphology from the surface of relief terraces. Knoll et al. found that vertical cylinders or parallel cylinders with necks preferentially form at the edge of relief terraces that comprise multiple layers of parallel cylinders on the flat region.^2,4^ Harrison et al. found a mixture of dots and perforations on the edge of relief terraces (islands) comprising multiple parallel cylinders.^42^ Ludwigs et al. found that parallel cylinders preferentially grew on the edge of relief terraces whose surface shows a morphology of perforated layers.^43^ van Dijk et al. found that cylinders form with either parallel or perpendicular orientations in terraced films.^44^ In that case, parallel cylinders form in thick regions and perpendicular cylinders form in thin regions. A mixture of perpendicular and parallel orientations usually appears as a morphology of isolated nanodots and parallel nanostripes in the images.

Nevertheless, Knorad et al. carefully re-examined the morphology of isolated nanodots and parallel nanostripes by combining imaging and plasma etching.^45^ Their study further clarified that such a morphology is displayed by the coexistence of parallel cylinders with and without necks. The study by Knorad et al. also points out that care needs to be taken to explore both surface and inner morphologies for thin films of BCPs. If an imaging technique is mainly used to probe the inner morphology of a BCP film, then etching is necessary. Our previous studies have demonstrated a variety of nanodomains in films of polystyrene-*block*-poly(methyl methacrylate) blended with a homopolystyrene (hPS) of different molecular weights at various weight fractions.^46−48^ The morphology, spatial ordering, and dimensions of the nanodomains vary with changing temperatures, film thicknesses, and blending compositions. For example, Hong et al. demonstrated the presence of perforated layers as a single phase in thin films of PS-*b*-PMMA-rich blends, regardless of the annealing temperature employed.^46^ With an increase in film thickness or temperature, these perforated layers often coexisted with double gyroids^46^ or parallel cylinders^49^ in thick films of PS-*b*-PMMA-rich blends. These complex phases, such as double gyroids or perforated layers, have been observed at the phase boundary between lamellae and cylinders.^50,51^ Perforated layers, identified as a long-lived metastable phase in bulk due to high packing frustration,^52^ can be stabilized through various blending approaches.^46−51,53^ Another strategy to enhance the stability of perforated layers is to reduce the film thickness.^2,54,55^ In our previous study, we found that not only do perforated layers form on the surface, but parallel cylinders also simultaneously grow in the interior during isothermal annealing at 230 °C in thick films of PS-*b*-PMMA-rich blends.^49^ However, the epitaxial relationship of spatial ordering between surface perforations and inner cylinders has not been thoroughly clarified. This study aims to address the epitaxial relationship of surface perforations with inner cylinders, particularly those oriented horizontally in the film interior. The current study aims to focus on how different thermal histories influence spatial ordering, orientations of the nanodomain, and morphologies of relief terraces (islands and holes) in thick films of PS-*b*-PMMA-rich blends.

## Experiments

Symmetric polystyrene-*block*-poly(methyl methacrylate) (*M*_n_^PS^ = 21 kg/mol and *M*_n_^PMMA^ = 21 kg/mol; *Đ* = 1.07) and homopolystyrene (hPS, *M*_n_ = 6.1 kg/mol; *Đ* = 1.05) were purchased from Polymer Source, Inc. The polymers were used as received without purification.

PS-*b*-PMMA and hPS were mixed in toluene under sonication (30 min) to prepare 5 wt % polymer solutions, in which the weight-fraction ratio of PS-*b*-PMMA to hPS was fixed at 75/25. Samples are briefly denoted as B_75_H_25_ blend films. For the sample code, B denotes PS-*b*-PMMA, H denotes hPS, and the subscripts denote blending weight fractions for PS-*b*-PMMA and hPS, respectively. Spin coating the 5 wt % solutions at 1000 rpm produced films with center regions having an average thickness of 282 ± 2 nm. Nevertheless, the periphery of the films inevitably forms thick beads. The thick beads are ascribed to surface tension by which spun materials accumulate at the substrate’s periphery, which cannot be avoided during spin coating.^56,57^ The thickness of the edge bead is much thicker than the thickness of the middle film.

Two different one-stage procedures of thermal annealing were employed on the as-spun films. As-spun films were directly annealed at 230 °C for 1 or 48 h after drying. A two-stage thermal annealing procedure was performed. As-spun films were soaked at 310 °C for 10 min to remove the history of spin coating and then annealed at 230 °C for 48 h. Our TGA measurements indicate that the PS-*b*-PMMA and hPS mainly degraded above 400 °C.^49^ All of the thermal treatments were performed in a vacuum furnace (Thermal Scientific, F79000). Thus, soaking at 310 °C did not significantly degrade the polymers.

The isothermally annealed films were characterized by grazing incident small-angle X-ray scattering (GISAXS) for structural analysis in a reciprocal space. GISAXS experiments were performed at beamline TPS 25A at the National Synchrotron Radiation Research Center (NSRRC) in Hsinchu. The dimension of an X-ray microbeam was approximately 5 μm^2^. Two-dimensional GISAXS patterns were recorded at an incident angle of α_i_ = 0.03° under an energy of 15 keV. A photon-counting area detector (Eiger X 16M) was used to record 2D GISAXS patterns with intensity distribution as a function of q_//_ and q_⊥_. q_//_ and q_⊥_ denote the vertical and horizontal components of the scattering vector, respectively.

Film thicknesses were measured by using an optical interferometer (Filmetrics, F20–UV). Relief terraces in the annealed B_75_H_25_ films were observed by an optical microscope (OM, Olympus, BX-BLA2) in reflection mode. Self-assembled nanodomains were observed with a field-emission scanning electron microscope (FE-SEM, Hitachi SU8200). Top-view images were recorded by SEM at 10 kV by collecting secondary electrons to observe the morphologies of self-assembled nanodomains. The films were fractured by a diamond knife to probe the inner morphologies. Fractured surfaces were characterized by SEM at a tilted angle of 25° to record side-view images. To increase the morphological contrast between PS and PMMA nanodomains, the top surfaces and fractured surfaces of films were exposed to oxygen plasma (oxygen plasma cleaner, Femto, FC111202) in different time intervals (15 and 30 s), by which PMMA nanodomains and short hPS chains can be quickly etched.^49^ Oxygen plasma etching was performed at a power of 90 W under a flow rate of oxygen gas of 10 sccm. Before purging oxygen gas, the chamber of oxygen plasma was vacuumed until the pressure had reached 10^–1^ mTorr. After oxygen plasma etching, the films were deposited on a thin layer (10 nm) of gold for SEM characterization. Fast Fourier transform (FFT) patterns were further analyzed on SEM images by Gwyddion software.^58^

## Results and Discussion

**Figure 1:**
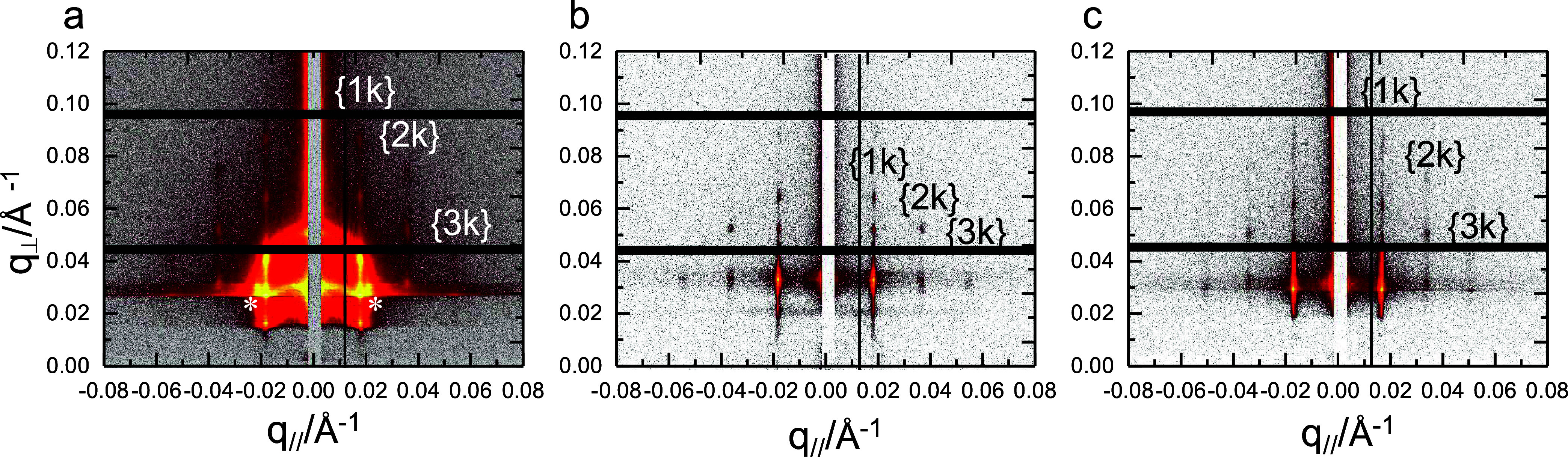
2D GISAXS patterns for three B_75_H_25_ films, which were subjected to one-stage thermal annealing of (a) 230 °C/1 h and (b) 230 °C/48 h and two-stage thermal annealing of (c) 310 °C/10 min and 230 °C/48 h. Arcs in (a) are highlighted by white asteroids.

Figure 1 shows the GISAXS patterns for three B_75_H_25_ films. Before GISAXS measurements, two different thermal annealing procedures were employed on as-spun films. As-spun films were subjected to isothermal annealing at 230 °C for 1 or 48 h after drying (i.e., one-stage thermal annealing procedure). Alternatively, spun films were first annealed at 310 °C (10 min) and then annealed at 230 °C (48 h) (two-stage procedure of thermal annealing). The designed thermal treatments allow us to test whether the obtained phases are in an equilibrium or kinetically trapped state. Kinetically trapped metastable phases change at different annealing histories, whereas equilibrium phases remain unchanged regardless of different thermal histories.

Figure 1 demonstrates fiber-like patterns for the annealed films. This result indicates that almost all nanodomains are preferentially oriented on the substrate. All diffractions can be assigned to scattering characteristics of parallel cylinders with hexagonal arrays. The q_//_ components of those diffractions locate at positions with a q_//_^{hk}^-to-q_//_^{1k}^ ratio of 1:2:3. The ratio is similar to the literature.^59,60^ Considering a c2mm symmetry assigned for deformed hexagonal arrays of parallel cylinders, *h* = *n* and *k* = 2*n* + 1, where *n* = 0, 1, 2, 3.^61^ This result indicates that the B_75_H_25_ films mainly formed parallel cylinders with distorted hexagonal arrays.

Note that Figure 1a shows two shoulder arcs (highlighted by white asteroids) near the first-order {1k} diffraction spots. This additional scattering is displayed only by the briefly annealed film at 230 °C. This feature is a kinetically trapped nanodomain with perpendicular orientation. Perpendicular orientation is unfavorable for cylinders on the hydrophilic surface of SiO_*x*_/Si, which selectively attracts the PMMA component. Our previous study demonstrated that such kinetically trapped nanodomains preferentially existed at the thick bead of the film because the kinetics of nanodomain ordering is slower at the edge bead than at the middle region.^62^Figure 1b,1c demonstrates no shoulder arcs for the films with prolonged annealing at 230 °C. Thus, the absence of shoulder arcs indicates that the kinetically trapped nanodomains at the thick bead of the film can be eliminated by prolonged annealing.

**Figure 2:**
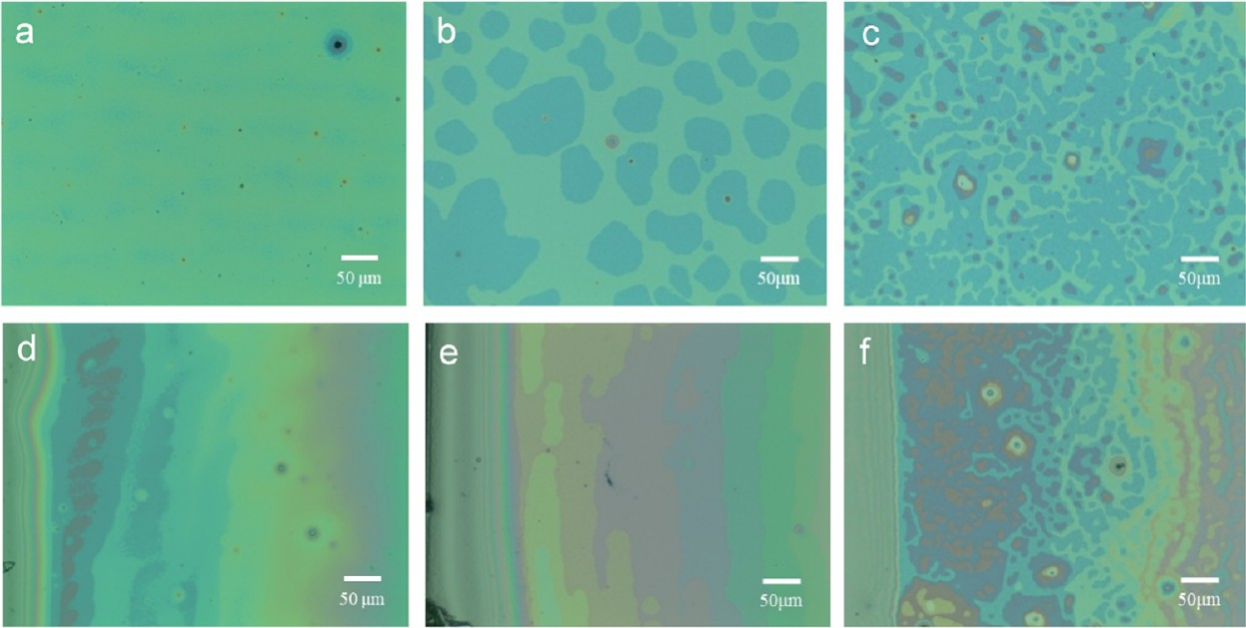
Optical microscopy images of three B_75_H_25_ films, which were subjected to one-stage thermal annealing of (a, d) 230 °C/1h and (b, e) 230 °C/48 h and two-stage thermal annealing of (c, f) 310 °C/10 min and 230 °C/48 h. OM images (a–c) were recorded in the center, whereas images (d–f) were recorded on the periphery.

We further performed OM measurements for morphological observations. Because OM provides information about local structures over small areas, we carefully recorded a series of OM images by scanning different areas for each film. Figure 2 shows representative OM images for the three B_75_H_25_ films. Figure 2 demonstrates three distinct morphologies. The first distinct morphology is that the thick beads of the three films formed relief terraces of multiple layers, whereas the middle regions formed relief terraces of two layers. Quantitatively analyzing the height profile of an atomic force microscopy (AFM) image demonstrates that each of the relief terraces is approximately 25.8 nm in height (for brevity, data are not shown). The height determined by AFM is comparable to the height of relief terraces in blend films determined by neutron reflectivity.^47,48^ Variations in thickness result in a color contrast in the OM images. Second, the edge of the relief terraces is diffuse for the B_75_H_25_ film that was shortly annealed at 230 °C (type-I terraces, Figure 2a,2d).

In comparison, the films with prolonged annealing formed sharp relief terraces (Figure 2b,2c,2e,2f). Third, one-stage prolonged thermal annealing produced well-defined steps (type-II terraces, Figure 2b,2c). In contrast, two-stage prolonged thermal annealing produced relief terraces with island coarsening (type-III terraces, Figure 2c,2f).

Scanning Electron Microscopy (SEM) characterization was performed to observe the morphology of the samples. It has been widely demonstrated that oxygen plasma etching removes PMMA at a high rate.^63−67^ To enhance the morphological contrast between PS and PMMA, the films were subjected to oxygen plasma etching at various intervals before SEM characterization. After 15 s of oxygen plasma etching, morphologies were observed through SEM characterization on different areas of a B_75_H_25_ film annealed at 230 °C for 1 h.

**Figure 3:**
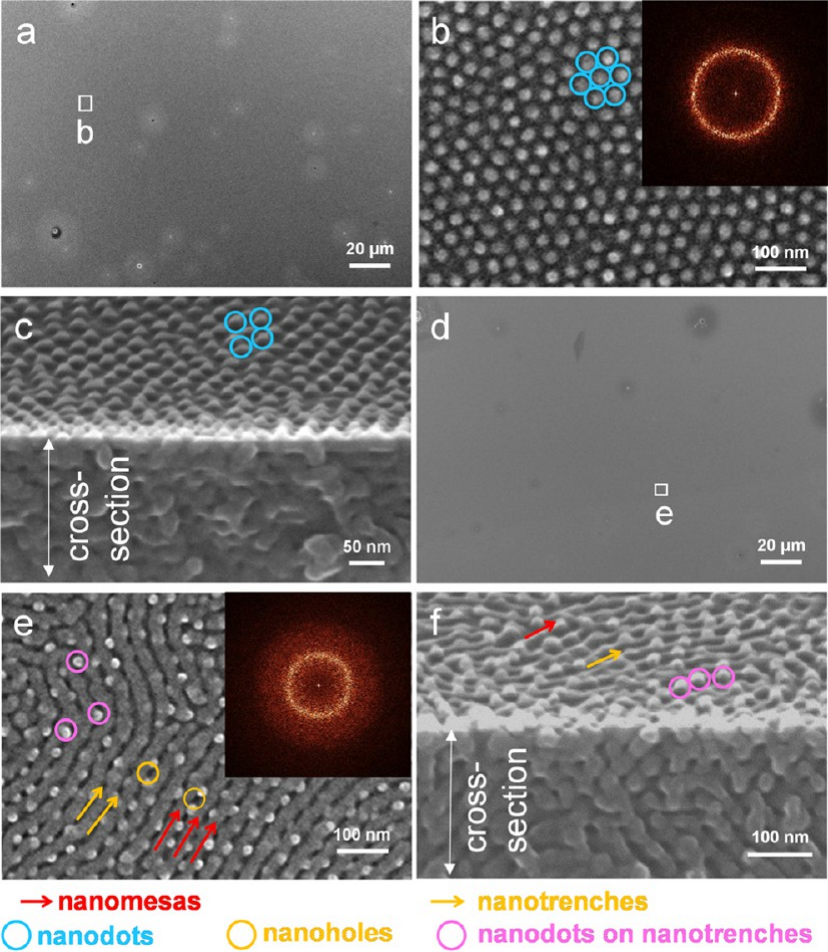
(a, b, d, e) Top-view and (c, f) side-view SEM images of a B_75_H_25_ film annealed at 230 °C for 1 h. Images (a)–(c) were recorded in the middle, whereas images (d)–(f) were recorded on the periphery. Insets are the FFT patterns of images (b) and (e). White boxes in (a) and (d) mark positions that were selected to record images (b) and (e), respectively. Before SEM characterization, the film had been exposed to oxygen plasma etching for 15 s. Nanodots and nanoholes are selectively highlighted by circles. Nanomesas and nanotrenches are highlighted by red and yellow arrows for visual guidance, respectively.

Figure 3 presents representative SEM images, showcasing distinct morphologies. First, relief terraces with diffuse edges, not easily discerned by top-view SEM (Figure 3a,3d), exhibit low resolution in the morphology, attributed to the low contrast between diffuse steps. Second, the thin center of the film reveals nanodot morphology (Figure 3b), while the thick periphery displays a coexistence of nanodots, nanoholes, nanomesas, and nanotrenches (Figure 3e). In the mixed morphology, nanodots appear to be positioned on top of the nanotrenches. Third, the FFT patterns of Figure 3b,3e reveal a powder-ring pattern, indicating that the in-plane nanodomains lack long-range order and form in-plane polygrains.

A noteworthy feature is that the nanodots are not PMMA cylinders that are oriented perpendicularly. Instead, the nanodots correspond to PS perforations. The rationale is that the removal of PMMA cylinders forms holes in a PS matrix,^68^ whereas the removal of a perforated PMMA layer leaves PS perforations,^46,47^ which can appear as nanodots. However, nanodots (Figure 3c) or their coexistence with nanoholes, nanomesas, and nanotrenches (Figure 3f) only form on the surface and do not extend through the entire thickness.

A cross-sectional view of perforated layers with a parallel orientation typically displays layer-by-layer packing. However, neither Figure 3c nor Figure 3f shows layer-by-layer packing along the film depth. Instead, it seems that the interior of the film forms parallel cylinders with short-range order, although the free surface is dominated by a skin layer of either nanodots (Figure 3c) or their coexistence with nanomesas, nanotrenches, and nanoholes (Figure 3f).

**Figure 4:**
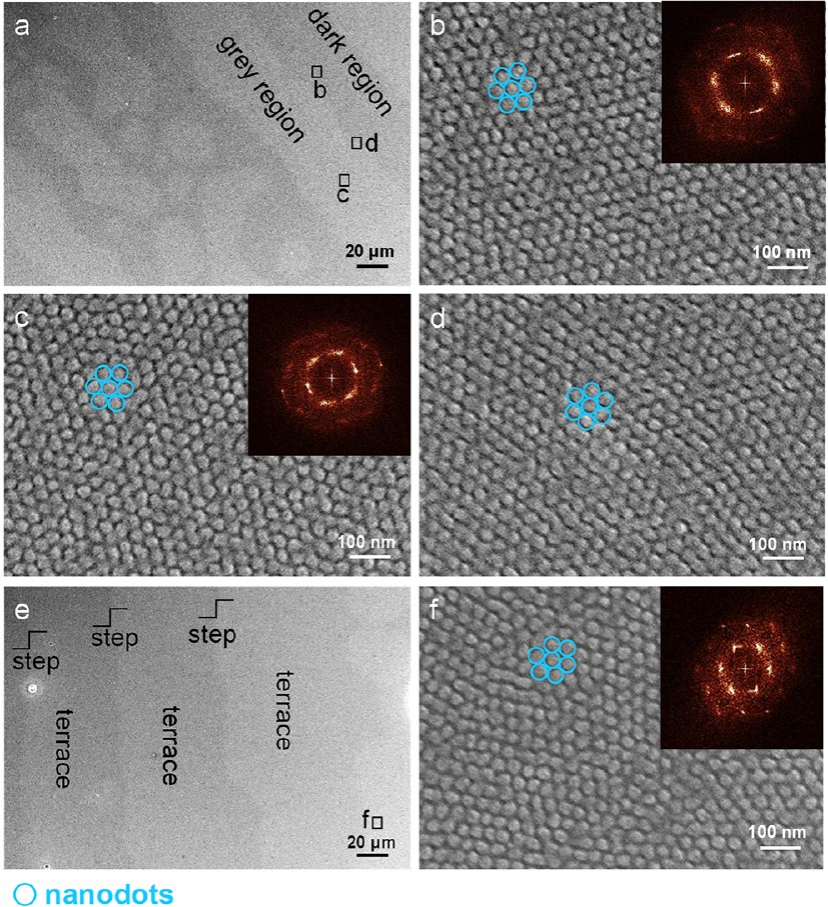
Top-view SEM images for a B_75_H_25_ film prolongedly annealed at 230 °C (48 h). Images (a–d) were recorded over different areas in the center, and images (e–f) were recorded over the periphery. The positions of recording images (b–d) and (f) are marked in images (a) and (e), respectively, for visual guidance. Insets are the FFT patterns. Before SEM characterization, the surface of the film had been exposed to oxygen plasma (15 s) for etching. Nanodots on the surface are selectively marked by blue circles for visual guidance.

Figure 4 shows top-view SEM images for B_75_H_25_ prolongedly annealed at 230 °C. Figure 4a shows the morphology of relief terraces with step edges for the center of the film prolongedly annealed at 230 °C, respectively. Regions forming relief terraces display dark, whereas terrace-free regions display gray. The color contrast is due to a height difference. Figure 4b−4d were obtained by zooming in a dark region, a gray region, and a border between the dark and gray regions, respectively. All of the high-magnification SEM images show a single morphology of nanodots (Figure 4b–d). No structural difference was found for the three regions. Furthermore, the nanodots are ordered with deformed hexagonal rather than regular hexagonal arrays. However, prolonged annealing can significantly improve the order of the nanodots (see the insets in Figure 4b,c).

The periphery shows a morphology of terraces with step edges (Figure 4e). Similarly, by zooming in on different periphery areas, we found that the periphery shows no structural difference. We observed only observed nanodots. Furthermore, interstep boundaries cannot be identified by zooming in on a border between two steps (Figure 4f).

**Figure 5:**
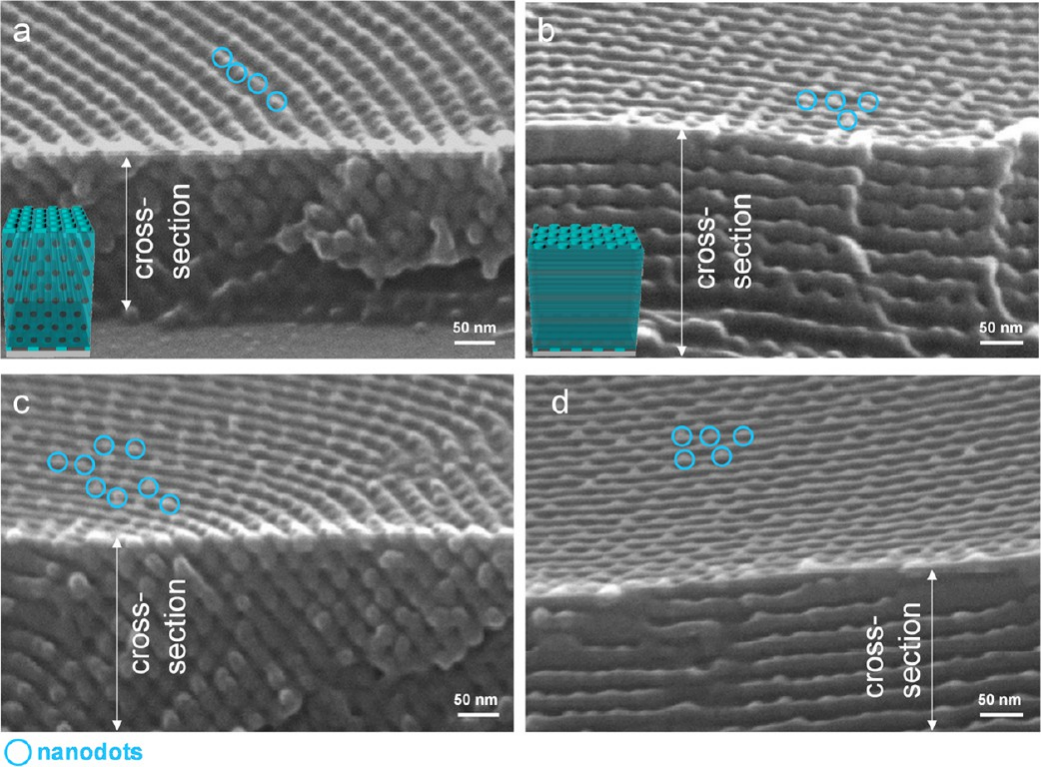
Side-view SEM images of the same film of Figure 4. Images (a) and (b) were recorded at the center, and images (c) and (d) were recorded at the periphery. Before SEM characterization, the film had been exposed to oxygen plasma etching for 15 s. Schemes inserted in (a) and (b) depict the coexistence of surface perforations and parallel cylinders in grains, each of which has a different orientation. Nanodots ordered in arrays on the surface are highlighted by blue circles for visual guidance.

Figure 5 shows side-view SEM images for the same blend film. For the side-view morphological observation, the film was fractured and then exposed to oxygen plasma for etching. Figure 5 suggests that the interior of the film should preferentially form parallel cylinders with deformed hexagonal arrays. Only the top surface and the substrate interface are dominated by perforations. It has been demonstrated that reducing film thickness can prevent two-phase coexistence and promote the exclusive formation of perforated layers.^5,46,55^ We further investigated the morphologies of a thin film with an initial thickness (*h*_i_) of approximately 80 nm. The thin film exclusively exhibited the formation of perforated layers (Figure S1). However, when the initial thickness exceeded that of three layers of parallel cylinders, perforated layers and parallel cylinders coexisted in thick films. In these thick films, perforated layers dominate the film surface, while parallel cylinders prevail in the interior.

This finding aligns with mesoscale modeling of phase behavior in thin films.^5^ The study by Horvat et al.^5^ demonstrated that surface fields at the polymer/air and polymer/substrate interfaces play a crucial role in the formation of perforated layers for films of a block copolymer (BCP) that primarily forms cylinders in bulk. An attractive surface or interface induces a discrepancy between the surface (interface) and the film interior. In our study, we propose that the formation of surface perforations is linked to an interplay between the surface field and spatial confinement. This interplay results in the prevalent existence of perforated layers on the surface of thick blend films or throughout the entire thickness of thin films.

**Figure 6:**
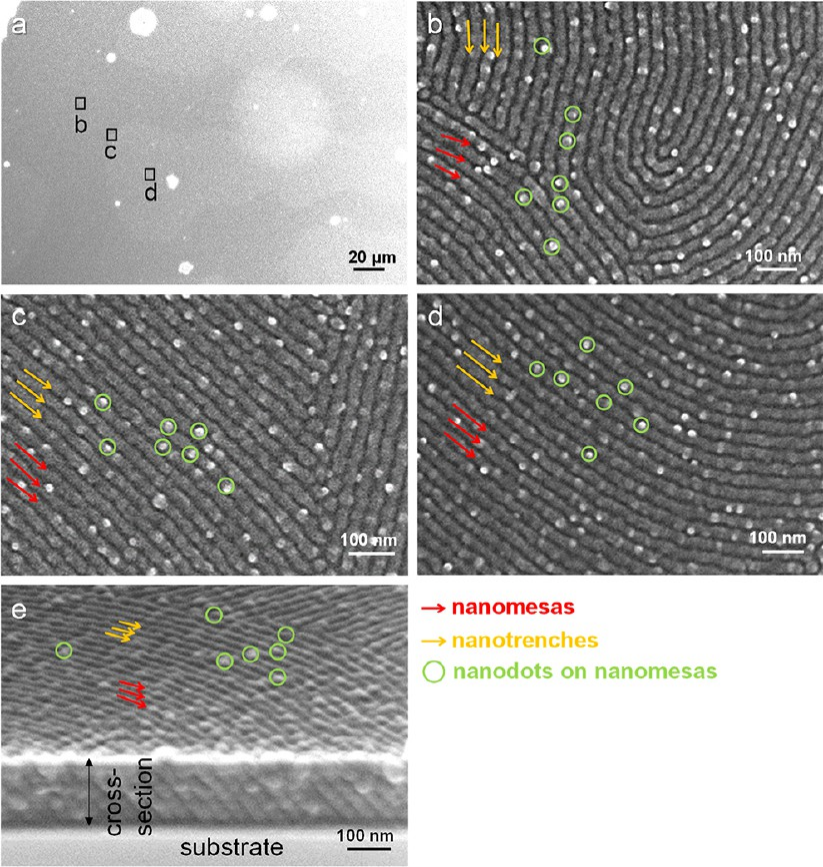
Top-view SEM images of (a) relief terraces, (b–d) top-view SEM images, and (e) side-view SEM image of parallel cylinders measured for a B_75_H_25_ film with prolonged annealing at 230 °C (48 h). Images (b)–(d) were recorded over three different areas, which are marked by boxes in image (a). Before SEM characterization, the film had been etched by oxygen plasma exposure (30 s). In (b–e), some nanodots, nanomesas, and nanotrenches are selectively highlighted for visual guidance.

Oxygen plasma exposure was performed for 30 s to probe parallel cylinders inside the film. After oxygen plasma exposure of 30 s, the top layer of surface perforations was removed (Figure 6). Thus, only morphologies of parallel cylinders are observed. The removal of parallel PMMA cylinders produced nanotrenches (indicated by yellow arrows) alternating with nanomesas (indicated by red arrows) composed of the PS component. Scrutiny of Figure 6b–d demonstrates that the parallel cylinders grew with abundant defects (dislocations and disclinations). Note that Figure 6c was recorded from a boundary between two terraces. Figure 6c indicates that parallel cylinders could grow across different terraces. Nevertheless, the boundary cannot be identified because of the low contrast in the presence of abundant defects. Note that the surface of the film is partially covered by nanodots located on the nanomesas. Unlike the nanodots packed with hexagonal arrays, the nanodots on nanomesas lack long-range ordering. Thus, the nanodots located on nanomesas may be attributed to incomplete removal of the skin layer or surface roughening induced by oxygen plasma etching.^63,64^

**Figure 7:**
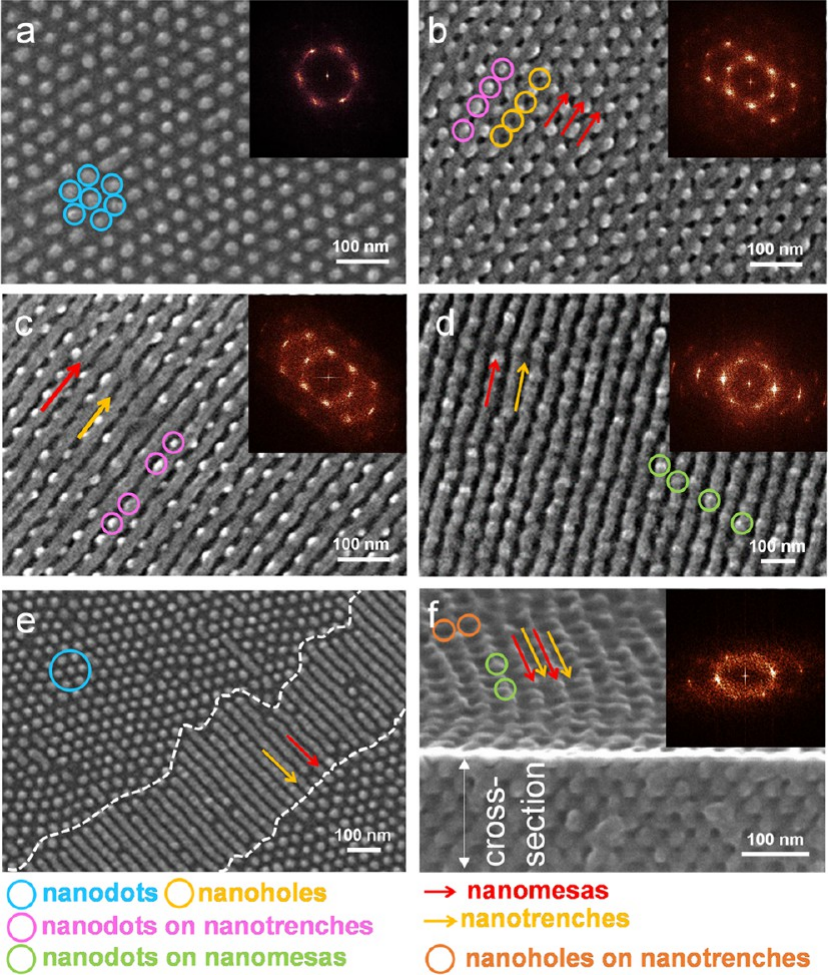
(a–e) Top-view and (f) side-view SEM images for a B_75_H_25_ film, which was soaked at 310 °C (10 min) and then prolongedly annealed at 230 °C (48 h). The top-view SEM images were recorded at different areas: (a–f) center and (b) periphery. Inserts in (a)–(d) and (f) are FFT patterns. Nanodots and nanoholes are highlighted by circles, respectively. Nanomesas and nanotrenches are highlighted by red and yellow arrows for visual guidance, respectively.

SEM characterization was also performed on the film that was soaked at 310 °C (10 min) and then isothermally annealed at 230 °C (48 h). Figures 7a–7e and S2 show representative top-view SEM images collected at different positions for the film. A collection of top-view SEM images displays four prominent morphologies: (i) hexagonal arrays of nanodots (selectively highlighted in Figure 7a); (ii) alternate arrays of smooth nanomesas and ordered nanodots (Figure 7b,7c); (iii) alternate arrays of rugged nanomesas and expanded nanoholes (Figure 7d); and (iv) coexistence of nanomesas, nanotrenches, and ordered nanodots (Figure 7e). Images in Figure 7a–7d were frequently present on the surface of terraces due to the nonuniform thickness throughout the film. Note that for the type-ii morphology, each set of nanodots was sandwiched by two nanomesas (Figure 7b,7c). Furthermore, the expanded nanoholes and the rugged surface of nanomesas can be discerned from the side-view image of the type-(iii) morphology (Figure 7f).

Furthermore, the ordered nanodots coexist alternately with nanoholes along regions confined by two neighboring nanomesas (Figure 7b). The image in Figure 7e was frequently observed when SEM characterization was performed on a border between two terraces that preferentially grow nanodots on their surfaces. As a result, each terrace’s flat region shows a nanodot morphology. In contrast, the regions between two neighboring terraces display a morphology of the coexistence of nanomesas and nanotrenches (Figure 7e). Furthermore, we found that the nanodots on different terraces epitaxially pack with the nanomesas and nanotrenches (Figure 7e).

The FFT analysis of Figure 7a displays six diffraction spots. The spots pack into a hexagonal array. However, the hexagonal array is not perfectly regular and is slightly deformed. The FFT analysis of Figure 7b,7c displays two sets of diffraction spots. The first set of spots is diffracted by the nanomesas and nanotrenches packed periodically in a series. The spots in the first set show intense intensity along the normal direction of the nanomesas and nanotrenches. The nanodots diffract the second series of spots. The nanodots alternate with the nanomesas and have a c2mm symmetry. In addition to the first-order diffraction spots, high-order diffraction spots are also present.

Nevertheless, the presence of high-order diffraction spots is anisotropic. Thus, the pattern resembles three hexagonal arrays of six diffractions superimposed together. The superimposition of three hexagonal arrays of six diffractions should be ascribed to the graphoepitaxy of alternate nanodots and nanoholes inside nanostripes (Figure 7b,7c).

An FFT analysis of Figure 7d displays a similar superimposition pattern of three hexagonal arrays with six diffractions. This pattern is unexpected. At a glance, Figure 7d shows a nanostripe-rich morphology. Thus, the nanostripes (i.e., nanomesas and nanotrenches) should have contributed only intense diffractions along their normal direction. If there are no nanodomains inside regions sandwiched by any two neighboring nanomesas, there should be no diffraction spots that can be assigned to a c2mmsymmetry.

**Figure 8:**
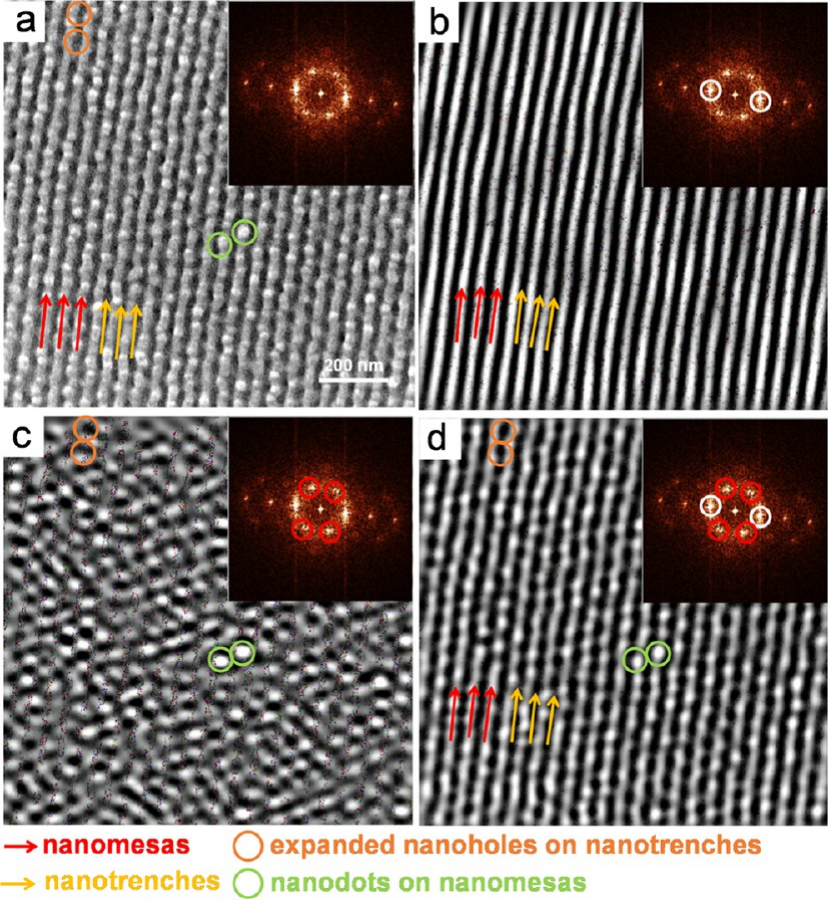
Detailed analysis of alternate arrays of nanomesas and expanded nanoholes for Figure 7d. (a) High-magnification top-view SEM image and corresponding FFT pattern and (b–d) reconstructed images by the invert fast Fourier transform of selected diffraction spots. The selected diffraction spots are marked by white and red circles in the FFT patterns of images (b–d), respectively. Nanomesas, nanotrenches, expanded nanoholes, and disordered nanodots are selectively marked by red arrows, yellow arrows, orange circles, and green circles for visual guidance, respectively.

A high-magnification view of the morphology presented in Figure 7d is illustrated in Figure 8a, revealing that the nanotrenches, alternating with rugged nanomesas, exhibit expanded nanoholes. The expanded nanoholes are distinctly highlighted by orange circles in Figure 8a. Additionally, Figure 8a illustrates that the nanomesas possess a rugged surface, selectively emphasized by green circles. The uneven surface of the nanomesas appears to result from nonuniform oxygen plasma etching. The expanded nanoholes contribute to unexpected diffraction spots, as is evident in the inset in Figure 8a. This deduction is based on reconstructed images obtained through the inverted Fourier transform of selected spots.

An image reconstructed from the spots marked by white circles in the FFT pattern (see the inset in Figure 8b) displays a series of alternating white and black nanostripes (Figure 8b). As compared to Figure 8a, the white and black nanostripes correspond to nanomesas and nanotrenches, respectively. Furthermore, an image formed from the spots identified by red circles in the FFT pattern (refer to the inset in Figure 8c) reveals the morphology of alternating white and black nanodomains. As compared to Figure 8a, the white and black nanodomains correspond to disordered nanodots and expanded nanoholes, respectively. The arrays of white and black nanodomains exhibit mirror and glide symmetry, denoted as c2mm symmetry. A superimposition of these two reconstructed images (Figure 8d) precisely reproduces the morphology presented in Figure 8a.

**Figure 9:**
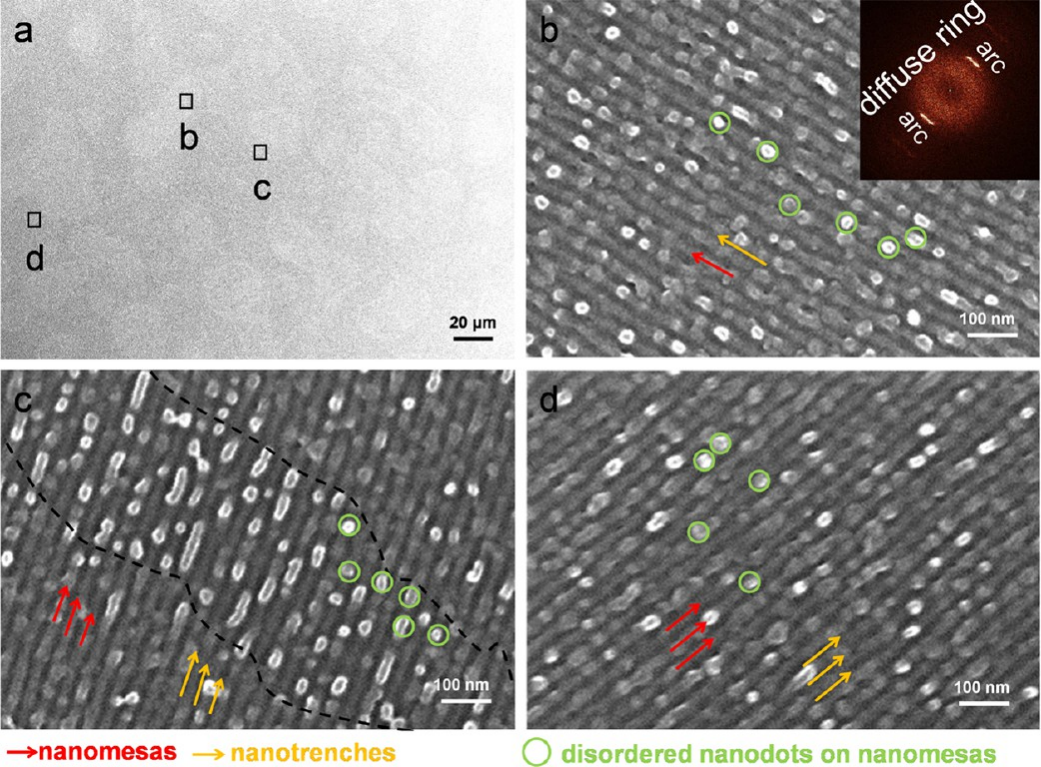
(a) Low- and (b–d) high-magnification top-view SEM images for a B_75_H_25_ film, which was soaked at 310 °C (10 min) and then prolongedly annealed at 230 °C (48 h). Images (b)–(d) were recorded on three different terraces, respectively. The recorded areas are labeled in image (a). Image (c) was obtained for a border between relief terraces. Oxygen plasma etching was performed for 30 s. Disordered nanodots on the nanomesas are selectively highlighted by green circles. Nanomesas and nanotrenches are indicated by red and yellow arrows for visual guidance, respectively.

The unique SEM images and FFT patterns are due to an etching effect from a short exposure to oxygen plasma. Figure 9 shows top-view SEM images after the terraced films were exposed to oxygen plasma etching for 30 s. The ordered arrays of nanodots are lost. Even nanoholes totally disappear. Instead, alternate nanomasas and nanotrenches are left. The nanotrenches correspond to the removal of PMMA cylinders, whereas the nanomesas correspond to the PS matrix. The nanomesas are covered with abundant disordered nanodots. The corresponding FFT pattern (inset in Figure 9b) shows two intense arcs in series along the radius direction of the parallel cylinders. Note the presence of a diffuse scattering ring in the FFT pattern. This diffuse ring is likely associated with the formation of disordered nanodots on nanomesas.

The disordered nanodots on nanomesas are distinct from the debris of surface perforations, as surface perforations exhibit ordered arrays. The formation of disordered nanodots on nanomesas is attributed to nonuniform etching induced by oxygen plasma^63,64^ Previous studies have reported that oxygen plasma etching involves both chain scission and cross-linking processes.^63^ Upon exposure to oxygen plasma, chain scission predominantly governs the etching of PMMA, while both chain scission and cross-linking simultaneously influence the etching of PS. Consequently, PMMA can be etched at a significantly higher rate than PS. The etching selectivity of PMMA to PS is influenced by various factors such as gas types, gas mixtures, and bias voltages.^63−68^ For instance, the etching selectivity of PMMA to PS reaches its maximum, approximately 3.9, when plasma etching is conducted in an Ar environment.^67^ In contrast, this selectivity can be reduced to the range of 1.3–2.1 when plasma etching is performed in O_2_^63−67^

Furthermore, if cross-linking rates are comparable to chain scission rates, surface roughening may occur due to polymer aggregation induced by cross-linking under energetic ion bombardment.^63^ We propose that the formation of disordered nanodots on nanomesas is associated with surface roughening induced by the interplay between chain scission and cross-linking^63^

Estimating the thickness of surface perforations necessitates quantifying the etching rates for PS. This approach is justified by two reasons: first, surface perforations exclusively consist of the PS block, and second, as PMMA has a higher etch rate than PS, the etching rate of the PS block predominantly influences variations in film thickness with exposure times under oxygen plasma. Our previous study revealed that an hPS of 17 kg/mol had an etch rate of 0.29 nm/s, while an hPS of 6.1 kg/mol had an etch rate of 0.97 nm/s under the same conditions of oxygen plasma etching.^49^ Considering the higher molecular weight of the PS block compared with 17 kg/mol, the etching rate of the PS-*b*-PMMA/hPS blend films is primarily determined by the etching rate of the PS block. Figure 9 illustrates that the layer of surface nanodots can be completely removed within 30 s of oxygen plasma exposure. This result implies that the thickness of the skin layer should approximately range from 8.7 to 29.1 nm, with the lower thickness based on the lower etching rate and the higher thickness based on the higher etching rate.

A comparison of Figures 6 and 9 demonstrates that two prominent features need further attention for nanomesas and nanotrenches. First, abundant in-plane dislocations and disclinations coexist in the film that was prolongedly annealed at 230 °C (Figure 6). As a result, the terraces are polygrains. Due to low contrast in the same morphology of nanotrenches alternate with nanomesas, the boundaries between terraces are challenging to identify by SEM. A boundary between two terraces was carefully examined for the film with prolonged one-stage annealing. The boundary also displays a morphology of polygrain texture, for which many grains are randomly oriented to each other. In contrast, such a polygrain texture was not easily found for the film with prolonged two-stage annealing (Figure 9). This result indicates that prolonged two-stage annealing significantly suppresses in-plane dislocations and disclinations.

Second, the suppression of defects results in the formation of monograin terraces. As a result, nanomesas and nanotrenches can grow across different terraces without defects (Figure 9c). The boundaries between different terraces are not due to the lateral packing of randomly oriented grains of parallel cylinders. There should be out-of-plane dislocations along the depth of the film.

**Figure 10:**
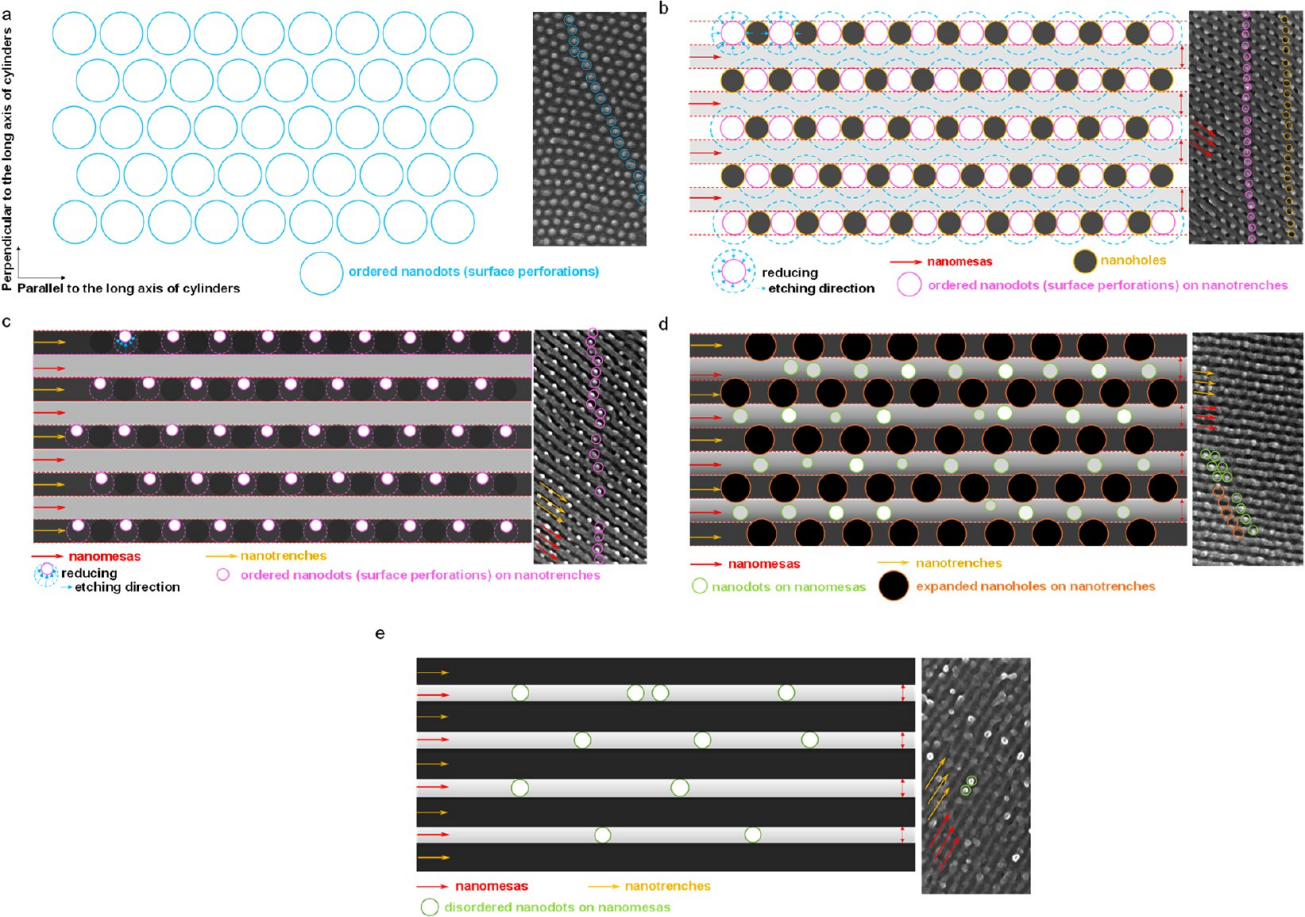
Schema of structural evolutions proposed for surface perforations during oxygen plasma for three morphologies: (a) hexagonal arrays of nanodots, (b, c) alternate arrays of nanomesas and nanodots on nanotrenches, (d) alternate arrays of nanomesas and expanded nanoholes on nanotrenches, and (e) alternate stacks of nanomesas and nanotrenches. For the sake of brevity, parallel cylinders of multiple layers buried underlying the surface perforations are omitted. Blue, pink, and green circles represent nanodots with varying spatial arrangements arising from etched PS blocks, respectively. Orange and yellow circles denote nanoholes as a result of removed PMMA blocks. Yellow and red arrows denote nanotrenches and nanomesas, respectively. The schemes are based on SEM images.

Figure 10 illustrates the structural evolution of a skin layer of perforations above parallel cylinders under oxygen plasma etching at various intervals. The schemes illustrated in Figure 10 are based on morphological observations from SEM characterization. Before oxygen plasma etching, the surface of terraces is dominated by a thin layer of perforations. The hexagonal arrays of nanodots indicate that only the skin layer is etched by short exposure to oxygen plasma, by which the PMMA component is etched. As a result, only PS perforations are left on the surface (Figure 10a). The arrays of PS perforations contribute to a six-spot FFT pattern. Since the arrays of the PS perforations orient epitaxially with the arrays of parallel cylinders underlying them, the six-spot FFT pattern is not regularly hexagonal but slightly deformed. The interperforation distance slightly expands perpendicular to the radius direction of parallel cylinders but shrinks along the radius direction of parallel cylinders (Figure 10a).

Oxygen plasma etching is isotropic but nonuniform. Because the annealed films have a nonuniform thickness, some regions experience a high dose of oxygen plasma. Thus, the extent of surface etching is high for regions with high doses of oxygen plasma. Once oxygen plasma etching reaches PMMA cylinders, surface etching becomes faster at PMMA-dominated domains than at PS-dominated ones. The highly etched regions produce nanoholes. This etching contrast causes alternate arrays between nanodots and nanoholes in a row; all orient epitaxially with the nearest cylinders (Figure 10b,10c). Because of the epitaxial packing, each row of alternate arrays between nanodots and nanoholes is sandwiched by two neighboring nanomesas. As a result, the FFT pattern of the morphology displays three hexagonal arrays of six diffraction spots.

Furthermore, the skin layer of perforations acts as a mask, for which underlying regions covered by PS perforations have a higher etching rate than regions free from PS perforations. This masking effect also results in the formation of expanded nanoholes in the next layer (Figure 10d). Prolonged oxygen plasma exposure (30 s) removes PS perforations. Without the skin layer, parallel PMMA cylinders and the PS matrix were exposed to the oxygen plasma. As a result, alternately nanomesas and nanotrenches are present (Figure 10e). The nanotrenches are due to the removal of PMMA cylinders. The nanomesas correspond to PS because PS is etched at a low rate.^63−68^ The disordered nanodots on nanomesas are due to surface roughening induced by prolonged oxygen plasma etching.^63^

The formation of surface perforations on top of parallel cylinders is highly dependent on the contents and molecular weights of hPS. For instance, even slight adjustments in the content of hPS with a molecular weight of *M*_n_ = 6.1 kg/mol in blend films can suppress surface perforations.^46,49^ In B_80_H_20_ films, lamellae emerge as the dominant phase.^46^ In contrast, in thick films of B_70_H_30_ and B_50_H_50_, parallel cylinders grow uniformly throughout the entire film thickness.^49^ This observation suggests that surface perforations represent an intermediate phase between lamellae and cylinders and are present within a narrow compositional range.

Furthermore, our previous study demonstrated that utilizing short chains of *M*_n_ = 2.8 kg/mol and long chains of *M*_n_ = 17 kg/mol for blend films tended to produce hexagonally perforated layers throughout the entire thickness at the same B_75_H_25_ ratio.^62^ In essence, the presence of parallel cylinders was completely inhibited when both short and long hPS chains were incorporated in the preparation of B_75_H_25_ blend films. For the case of employing hPS chains of *M*_n_ = 17 kg/mol in B_75_H_25_ blend films, the stabilization of hexagonally perforated layers is attributed to the local segregation of long chains into hot-spot regions within self-assembled nanodomains (referred to as dry brushes) to alleviate packing frustration. This mechanism has been extensively demonstrated by Matsen et al.^50−52^ Nevertheless, it remains unclear why the use of short chains of *M*_n_ = 2.8 kg/mol could also suppress parallel cylinders while stabilizing hexagonally perforated layers. This result is unexpected because short chains should be uniformly distributed within self-assembled nanodomains (referred to as wet brushes); thus, local segregation to fill hot-spot regions should not occur for short chains. It is plausible that the addition of low-molecular-weight species such as solvents introduces additional compressibility to solvated domains, further alleviating the local volume balance constraint and reducing the frustration of medial packing.^69^ However, understanding how the frustration of medial packing occurs requires structural information on fine features of subdomain geometry,^70^ which falls outside the scope of our current study.

In comparison with our previous study,^62^ the current investigation demonstrates that surface perforations and parallel cylinders can coexist in B_75_H_25_ thick films prepared by blending hPS with *M*_n_ = 6.1 kg/mol with PS-*b*-PMMA. This comparison indicates that the stabilization of hexagonally perforated layers has no monochronic dependence on the molecular weight of hPS.

## Conclusions

B_75_H_25_ films were prepared by blending a weakly segregated, symmetric PS-*b*-PMMA with an hPS at a weight fraction of 75/25. At the weight fraction, the B_75_H_25_ films preferentially form perforations on the surface but parallel cylinders in the inner after thermally annealing at 230 °C. Due to incommensurability between initial thickness and interdomain distance, the annealed films inevitably form relief terraces (islands and holes). We have demonstrated three morphologies of relief terraces: (I) terraces with diffuse edges, (II) terraces with step edges, and (III) terraces with island coarsening. These morphologies on the micrometer scale are correlated with the orientation and ordering of nanodomains and topological defects. Type-I terraces were obtained by directly annealing as-spun films at 230 °C (1 h). Misaligned nanodomains with abundant defects tend to favor type-I terraces. Type-II terraces were obtained by directly annealing as-spun films at 230 °C (48 h), by which misaligned orientation and defects can be removed. Nevertheless, dislocations and disclinations still exist in certain contents. Their presence results in the formation of small monograins inside type-II terraces. Furthermore, defects can reduce the entropic penalty of surface perforations at borders between type-II terraces. Thus, perforations grow on the surface of type-II terraces and the borders between type-II terraces.

Type-III terraces were obtained by soaking at 310 °C (10 min) and then annealing at 230 °C (48 h). The prolonged, two-stage thermal annealing significantly improves the ordering of nanodomains by reducing the number of in-plane defects. Thus, type-III terraces comprised pseudomonograins (i.e., single-crystal-like grains) each. We postulate that out-of-plane dislocations are linked to the formation of type-III terraces. Furthermore, because of the improvement in nanodomain ordering, perforations grow only on the surface of type-III terraces. Perforations are prohibited at the borders of type-III terraces.

Besides the morphologies of terraces, we have demonstrated how surface perforations orient epitaxially on top of parallel cylinders in blend films. The epitaxial match between surface perforations and inner cylinders explains how structural evolution occurs during oxygen plasma etching. Several morphologies are observed at the nanometer scale for type-III terraces etched by oxygen plasma: nanodots with deformed hexagonal arrays, coexistence of nanodots and nanoholes alternated with nanostripes, and nanostripes. The nanoscale morphologies are due to different etching depths if oxygen plasma of various exposure periods is performed on films forming surface perforations on top of parallel cylinders.

## Supporting Information Available

OM and top-view SEM images of a thin B_75_H_25_ blend film annealed at 230 °C for 48 h (Figure S1) and top-view SEM images of FFT patterns of a B_75_H_25_ blend film after being soaked at 310 °C for 10 min and then annealed at 230 °C for 48 h (Figure S2) (PDF)

## References

[ref1] Mastroianni S. E.; Epps T. H. III Interfacial Manipulations: Controlling Nanoscale Assembly in Bulk, Thin Film, and Solution Block Copolymer Systems. Langmuir 2013, 29, 3864–3878. 10.1021/la304800t. 23406541

[ref2] Knoll A.; Horvat A.; Lyakhova K. S.; Krausch G.; Sevink G. J. A.; Zvelindovsky A. V.; Magerle R. Phase Behavior in Thin Films of Cylinder-Forming Block Copolymers. Phys. Rev. Lett. 2002, 89, 035501 10.1103/PhysRevLett.89.035501. 12144400

[ref3] Huinink H. P.; Brokken-Zijp J. C. M.; van Dijk M. A.; Sevink G. J. A. Asymmetric Block Copolymers Confined in a Thin Film. J. Chem. Phys. 2000, 112, 2452–2462. 10.1063/1.480811.

[ref4] Knoll A.; Magerle R.; Krausch G. Phase Behavior in Thin Films of Cylinder-Forming ABA Block Copolymers: Experiments. J. Chem. Phys. 2004, 120, 1105–1116. 10.1063/1.1627324. 15267947

[ref5] Horvat A.; Lyakhova K. S.; Sevink G. J. A.; Zvelindovsky A. V.; Magerle R. Phase Behavior in Thin Films of Cylinder-Forming ABA Block Copolymers: Mesoscale. Modeling. J. Chem. Phys. 2004, 120, 1117–1126. 10.1063/1.1627325. 15267948

[ref6] Tsarkova L.; Horvat A.; Krausch G.; Zvelindovsky A. V.; Sevink G. A.; Magerle R. Defect Evolution in Block Copolymer Thin Films via Temporal Phase Transitions. Langmuir 2006, 22, 8089–8095. 10.1021/la0613530. 16952246

[ref7] Lyakhova K. S.; Sevink G. J. A.; Zvenlindovsky A. V.; Horvat A.; Magerle R. Role of Dissimilar Interfaces in Thin Films of Cylinder-Forming Block Copolymers. J. Chem. Phys. 2004, 120, 1127–1137. 10.1063/1.1632475. 15267949

[ref8] Huinink H. P.; Van Dijk M. A.; Brokken-Zijp J. C. M.; Sevink G. J. A. Surface-Induced Transitions in Thin Films of Asymmetric Diblock Copolymers. Macromolecules 2001, 34, 5325–5330. 10.1021/ma000015h.

[ref9] Zettl U.; Knoll A.; Tsarkova L. Effect of Confinement on the Mesoscale and Macroscopic Swelling of Thin Block Copolymer Films. Langmuir 2010, 26, 6610–6617. 10.1021/la903922y. 20027996

[ref10] Limary R.; Green P. F. Dewetting Instabilities in Thin Block Copolymer Films: Nucleation and Growth. Langmuir 1999, 15, 5617–5622. 10.1021/la981693o.

[ref11] Müller-Buschbaum P.; Maurer E.; Bauer E.; Cubitt R. Surface versus Confinement Induced Morphology Transition in Triblock Copolymer Films: A Grazing Incidence Small Angle Neutron Scattering Investigation. Langmuir 2006, 22, 9295–9303. 10.1021/la061455q. 17042545

[ref12] Müller-Buschbaum P.; Schulz L.; Metwalli E.; Moulin J. F.; Cubitt R. Interface-Induced Morphology Transition in Triblock Copolymer Films Swollen with Low-Molecular-Weight Homopolymer. Langmuir 2009, 25, 4235–4242. 10.1021/la802471p. 18954149

[ref13] Sparnacci K.; Antonioli D.; Gianotti V.; Laus M.; Lupi F. F.; Giammaria T. J.; Seguini G.; Perego M. Ultrathin Random Copolymer-Grafted Layers for Block Copolymer Self-Assembly. ACS Appl. Mater. Interfaces 2015, 7, 10944–10951. 10.1021/acsami.5b02201. 25954979

[ref14] Perego M.; Lupi F. F.; Giammaria T. J.; Seguini G.; Vita F.; Francescangeli O.; Sparnacci K.; Antonioli D.; Gianotti V.; Laus M. Fine Tuning of Lithographic Masks through Thin Films of PS-b-PMMA with Different Molar Mass by Rapid Thermal Processing. ACS Appl. Mater. Interfaces 2014, 6, 7180–7188. 10.1021/am5003074. 24738855

[ref15] Ham S.; Shin C.; Kim E.; Ryu D. Y.; Jeong U.; Russell T. P.; Hawker C. J. Microdomain Orientation of PS-b-PMMA by Controlled Interfacial Interactions. Macromolecules 2008, 41, 6431–6437. 10.1021/ma8007338.

[ref16] Huang E.; Russell T. P.; Harrison C.; Chaikin P. M.; Register R. A.; Hawker C. J.; Mays J. Using Surface Active Random Copolymers to Control the Domain Orientation in Diblock Copolymer Thin Films. Macromolecules 1998, 31, 7641–7650. 10.1021/ma980705+.

[ref17] Huang E.; Pruzinsky S.; Russell T. P.; Mays J.; Hawker C. J. Neutrality Conditions for Block Copolymer Systems on Random Copolymer Brush Surfaces. Macromolecules 1999, 32, 5299–5303. 10.1021/ma990483v.

[ref18] Ryu D. Y.; Ham S.; Kim E.; Jeong U.; Hawker C. J.; Russell T. P. Cylindrical Microdomain Orientation of PS-b-PMMA on the Balanced Interfacial Interactions: Composition Effect of Block Copolymers. Macromolecules 2009, 42, 4902–4906. 10.1021/ma900110w.

[ref19] Ji S.; Liao W.; Nealey P. F. Block Cooligomers: A Generalized Approach to Controlling the Wetting Behavior of Block Copolymer Thin Films. Macromolecules 2010, 43, 6919–6922. 10.1021/ma1007946.

[ref20] Han E.; Stuen K. O.; Leolukman M.; Liu C. C.; Nealey P. F.; Gopalan P. Perpendicular Orientatio n of Domains in Cylinder-Forming Block Copolymer Thick Films by Controlled Interfacial Interactions. Macromolecules 2009, 42, 4896–4901. 10.1021/ma9002903.

[ref21] She M. S.; Lo T. Y.; Ho R. M. Long-Range Ordering of Block Copolymer Cylinders Driven by Combining Thermal Annealing and Substrate Functionalization. ACS Nano 2013, 7, 2000–2011. 10.1021/nn305725q. 23438409

[ref22] Nakatani R.; Takano H.; Chandra A.; Yoshimura Y.; Wang L.; Suzuki Y.; Tanaka Y.; Maeda R.; Kihara N.; Minegishi S.; Miyagi K.; Kasahara Y.; Sato H.; Seino Y.; Azuma T.; Yokoyama H.; Ober C. K.; Hayakawa T. Perpendicular Orientation Control Without Interfacial Treatement of RAFT-Synthesized High-χ Block Copolymer Thin Films with Sub-10 nm Features Prepared via Thermal Annealing. ACS Appl. Mater. Interfaces 2017, 9, 31266–31278. 10.1021/acsami.6b16129. 28304153

[ref23] Nakatani R.; Chandra A.; Uchiyama T.; Nabae Y.; Hayakawa T. Dynamic Ordering in High-χ Block Copolymer Lamellae Based on Cross-Sectional Orientational Alignment. ACS Macro Lett. 2019, 8, 1122–1127. 10.1021/acsmacrolett.9b00353. 35619441

[ref24] Yoshida K.; Tanaka S.; Yamamoto T.; Tajima K.; Borsali R.; Isono T.; Satoh T. Chain-End Functionalization with a Saccharide for 10 nm Microphase Separation: “Classical” PS-b-PMMA Versus PS-b-PMMA-Saccharide. Macromolecules 2018, 51, 8870–8877. 10.1021/acs.macromol.8b02069.

[ref25] Yoshida K.; Tian L.; Miyagi K.; Yamazaki A.; Mamiya H.; Yamamoto T.; Tajima K.; Isono T.; Satoh T. Facile and Efficient Modification of Polystyrene-block-poly(methyl methacrylate) for Achieving Sub-10 nm Feature Size. Macromolecules 2018, 51, 8064–8072. 10.1021/acs.macromol.8b01454.

[ref26] Wang H.-F.; Marubayashi H.; Jinnai H. A Kinetic Pathway of the Order-Order Transition from Hexagonally Packed Cylinder to Hexagonally Perforated Layer in Polystyrene-block-Poly(2-vinylpyridine) Using Time-Resolved 3D Transmission Electron Microtomography. Macromolecules 2023, 56, 1503–1513. 10.1021/acs.macromol.2c01849.

[ref27] Müller-Buschbaum P.; Schulz L.; Metwalli E.; Moulin J. F.; Cubitt R. Lateral Structures of Buried Interfaces in ABA-Type Triblock Copolymer Films. Langmuir 2008, 24, 7639–7644. 10.1021/la801539r. 18620445

[ref28] Han E.; Gopalan P. Cross-Linked Random Copolymer Mats as Ultrathin Nonpreferential Layers for Block Copolymer Self-Assembly. Langmuir 2010, 26, 1311–1315. 10.1021/la902483m. 19791776

[ref29] Peters R. D.; Yang X. M.; Kim T. K.; Sohn B. H.; Nealey P. F. Using Self-Assembled Monolayers Exposed to X-Rays to Control the Wetting Behavior of Thin Films of Diblock Copolymers. Langmuir 2000, 16, 4625–4631. 10.1021/la991500c.

[ref30] Peters R. D.; Yang X. M.; Kim T. K.; Nealey P. F. Wetting Behavior of Block Copolymers on Self-Assembled Films of Alkylchlorosiloxanes: Effect of Grafting Density. Langmuir 2000, 16, 9620–9626. 10.1021/la000822+.

[ref31] Bates C. M.; Strahan J. R.; Santos L. J.; Mueller B. K.; Bamgbade B. O.; Lee J. A.; Willson C. G.; et al. Polymeric Cross-Linked Surface Treatments for Controlling Block Copolymer Orientation in Thin Films. Langmuir 2011, 27, 2000–2006. 10.1021/la1042958. 21214210

[ref32] In I.; La Y. H.; Park S. M.; Nealey P. F.; Gopalan P. Side-Chain-Grafted Random Copolymer Brushes as Neutral Surfaces for Controlling the Orientation of Block Copolymer Microdomains In Thin Films. Langmuir 2006, 22, 7855–7860. 10.1021/la060748g. 16922574

[ref33] Choi K. I.; Kim T. H.; Lee Y.; Kim H.; Lee H.; Yuan G.; Koo J.; et al. Perpendicular Orientation of Diblock Copolymers Induced by Confinement between Graphene Oxide Sheets. Langmuir 2018, 34, 1681–1690. 10.1021/acs.langmuir.7b03991. 29293348

[ref34] An S.; Kim H.; Kim M.; Kim S. Photoinduced Modulation of Polymeric Interfacial Behavior Controlling Thin-Film Block Copolymer Wetting. Langmuir 2020, 36, 3046–3056. 10.1021/acs.langmuir.0c00266. 32151131

[ref35] Yang W. C.; Wu S. H.; Chen Y. F.; Nelson A.; Wu C. M.; Sun Y. S. Effects of the Density of Chemical Cross-links and Physical Entanglements of Ultraviolet-Irradiated Polystyrene Chains on Domain Orientation and Spatial Order of Polystyrene-block-Poly (methyl methacrylate) Nano-Domains. Langmuir 2019, 35, 14017–14030. 10.1021/acs.langmuir.9b02054. 31577149

[ref36] Matsen M. W.; Bates F. S. Unifying Weak- and Strong-Segregation Block Copolymer Theories. Macromolecules 1996, 29, 1091–1098. 10.1021/ma951138i.

[ref37] Hamley I. W. Ordering in Thin Films of Block Copolymers: Fundamentals to Potential Applications. Prog. Polym. Sci. 2009, 34, 1161–1210. 10.1016/j.progpolymsci.2009.06.003.

[ref38] Coulon G.; Russell T. P.; Deline V. R.; Green P. F. Surface-Induced Orientation of Symmetric, Diblock Copolymers: A Secondary Ion Mass-Spectrometry Study. Macromolecules 1989, 22, 2581–2589. 10.1021/ma00196a006.

[ref39] Grim P. C. M.; Nyrkova I. A.; Semenov A. N.; Brinke G. T.; Hadziioannou G. The Free Surface of Thin Diblock Copolymer Films:Experimental and Theoretical Investigations on the Formation and Growth of Surface Relief Structures. Macromolecules 1995, 28, 7501–7513. 10.1021/ma00126a030.

[ref40] Kim S.; Bates C. M.; Thio A.; Cushen J. D.; Ellison C. J.; Willson C. G.; Bates F. S. Consequences of Surface Neutralization in Diblock Copolymer Thin Films. ACS Nano 2013, 7, 9905–9919. 10.1021/nn403616r. 24131385

[ref41] Maher M. J.; Self J. L.; Stasiak P.; Blachut G.; Ellison C. J.; Matsen M. W.; Bates C. M.; Willson C. G. Structure, Stability, and Reorganization of 0.5 L_0_ Topography in Block Copolymer Thin Films. ACS Nano 2016, 10, 10152–10160. 10.1021/acsnano.6b05390. 27787994

[ref42] Harrison C.; Park M.; Chaikin P.; Register R. A.; Adamson D. H.; Yao N. Depth Profiling Block Copolymer Microdomains. Macromolecules 1998, 31, 2185–2189. 10.1021/ma9716037.

[ref43] Ludwigs S.; Schmidt K.; Stafford C. M.; Amis E. J.; Fasolka M. J.; Karim A.; Magerle R.; Krausch G. Combinatorial Mapping of the Phase Behavior of ABC Triblock Terpolymers in Thin Films: Experiments. Macromolecules 2005, 38, 1850–1858. 10.1021/ma049048d.

[ref44] van Dijk M. A.; van den Berg R. Ordering Phenomena in Thin Block Copolymer Films Studied Using Atomic Force Microscopy. Macromolecules 1995, 28, 6773–6778. 10.1021/ma00124a011.

[ref45] Konrad M.; Knoll A.; Krausch G.; Magerle R. Volume Imaging of an Ultrathin SBS Triblock Copolymer Film. Macromolecules 2000, 33, 5518–5523. 10.1021/ma992057f.

[ref46] Hong J. W.; Chang J. H.; Chang I. C. Y.; Sun Y. S. Phase Behavior in Thin Films of Weakly Segregated Block Copolymer/Homopolymer Blends. Soft Matter 2021, 17, 9189–9197. 10.1039/D1SM01005K. 34586138

[ref47] Hong J. W.; Chang J. H.; Hung H. H.; Liao Y. P.; Jian Y. Q.; Chang I. C. Y.; Huang T. Y.; Nelson A.; Lin I. M.; Chiang Y. W.; Sun Y. S. Chain Length Effects of Added Homopolymers on the Phase Behavior in Blend Films of a Symmetric, Weakly Segregated Polystyrene-block-poly(methyl methacrylate). Macromolecules 2022, 55, 2130–2147. 10.1021/acs.macromol.1c02167.

[ref48] Hong J. W.; Jian Y. Q.; Liao Y. P.; Hung H. H.; Huang T. Y.; Nelson A.; Tsao I. Y.; Wu C. M.; Sun Y. S. Distributions of Deuterated Polystyrene Chains in Perforated Layers of Blend Films of a Symmetric Polystyrene-block-poly(methyl methacrylate). Langmuir 2021, 37, 13046–13058. 10.1021/acs.langmuir.1c02132. 34696591

[ref49] Sun Y.-S.; Jian Y.-Q.; Yang S.-T.; Chen C.-Y.; Lin J.-M. Morphologies of Surface Perforations and Parallel Cylinders Coexisting in Terraced Films of Block Copolymer/Homopolymer Blends with Oxygen Plasma Etching. Langmuir 2023, 39, 16284–16293. 10.1021/acs.langmuir.3c01784. 37934122

[ref50] Matsen M. W. Stabilizing New Morphologies by Blending Homopolymer with Block Copolymer. Phys. Rev. Lett. 1995, 74, 4225 10.1103/PhysRevLett.74.4225. 10058447

[ref51] Matsen M. W. Phase Behavior of Block Copolymer/Homopolymer Blends. Macromolecules 1995, 28, 5765–5773. 10.1021/ma00121a011.

[ref52] Matsen M. W.; Bates F. S. Origins of Complex Self-Assembly in Block Copolymers. Macromolecules 1996, 29, 7641–7644. 10.1021/ma960744q.

[ref53] Nowak S. R.; Tiwale N.; Doerk G. S.; Nam C.-Y.; Black C. T.; Yager K. G. Responsive Blends of Block Copolymers Stabilize the Hexagonally Perforated Lamellae Morphology. Soft Matter 2023, 19, 2594–2604. 10.1039/D3SM00142C. 36947412

[ref54] Park I.; Park S.; Park H. W.; Chang T.; Yang H.; Ryu C. Y. Unexpected Hexagonally Perforated Layer Morphology of PS-b-PMMA Block Copolymer in Supported Thin Film. Macromolecules 2006, 39, 315–318. 10.1021/ma0515937.

[ref55] Jung J.; Park H.-W.; Lee S.; Lee H.; Chang T.; Matsunaga K.; Jinnai H. Effect of Film Thickness on the Phase Behaviors of Diblock Copolymer Thin Film. ACS Nano 2010, 4, 3109–3116. 10.1021/nn1003309. 20499924

[ref56] Shiratori S.; Kubokawa T. Double-Peaked Edge-Bead in Drying Film of Solvent-Resin Mixtures. Phys. Fluids 2015, 27, 102105 10.1063/1.4934670.

[ref57] Junisu B. A.; Sun Y.-S. Hierarchical Surface Instability in Polymer Films. Langmuir 2023, 39, 15249–15259. 10.1021/acs.langmuir.3c01936. 37862459

[ref58] Nečas D.; Klapetek P. Gwyddion: An Open-Source Software for SPM Data Analysis.. Open Phys. 2012, 10, 181–188. 10.2478/s11534-011-0096-2.

[ref59] Lee B.; Park I.; Yoon J.; Park S.; Kim J.; Kim K. W.; Chang T.; Ree M. Structural Analysis of Block Copolymer Thin Films with Grazing Incidence Small-Angle X-ray Scattering. Macromolecules 2005, 38, 4311–4323. 10.1021/ma047562d.

[ref60] Saito I.; Miyazaki T.; Yamamoto K. Depth-Resolved Structure Analysis of Cylindrical Microdomain in Block Copolymer Thin Film by Grazing-Incidence Small-Angle X-ray Scattering Utilizing Low-Energy X-rays. Macromolecules 2015, 48, 8190–8196. 10.1021/acs.macromol.5b01883.

[ref61] Hsu C. H.; Yue K.; Wang J.; Dong X. H.; Xia Y.; Jiang Z.; Thomas E. L.; Cheng S. Z. D. Thickness-Dependent Order-to-Order Transitions of Bolaform-like Giant Surfactant in Thin Films. Macromolecules 2017, 50, 7282–7290. 10.1021/acs.macromol.7b01598.

[ref62] Sun Y.-S.; Liao Y.-P.; Hung H.-H.; Chiang P.-H.; Su C.-J. Molecular-Weight Effects of a Homopolymer on the AB- and ABC-Stacks of Perforations in Block Copolymer/Homopolymer Films. Soft Matter 2024, 20, 609–620. 10.1039/D3SM01249B. 38131364

[ref63] Ting Y.-H.; Liu C.-C.; Park S.-M.; Jiang H.; Nealey P. F.; Wendt A. E. Surface Roughening of Polystyrene and Poly (Methyl Methacrylate) in Ar/O_2_ Plasma Etching. Polymers 2010, 2, 649–663. 10.3390/polym2040649.

[ref64] Ting Y.-H.; Park S.-M.; Liu C.-C.; Liu X.; Himpsel F.; Nealey P. F.; Wendt A. E. Plasma Etch Removal of Poly (Methyl Methacrylate) in Block Copolymer Lithography. J. Vac. Sci. Technol., B 2008, 26, 1684–1689. 10.1116/1.2966433.

[ref65] Liu C.-C.; Nealey P. F.; Ting Y.-H.; Wendt A. E. Pattern Transfer Using Poly (Styrene-Block-Methyl Methacrylate) Copolymer Films and Reactive Ion Etching. J. Vac. Sci. Technol., B 2007, 25, 1963–1968. 10.1116/1.2801884.

[ref66] Farrell R. A.; Petkov N.; Shaw M. T.; Djara V.; Holmes J. D.; Morris M. A. Monitoring PMMA Elimination by Reactive Ion Etching from a Lamellar PS-b-PMMA Thin Film by ex Situ TEM Methods. Macromolecules 2010, 43, 8651–8655. 10.1021/ma101827u.

[ref67] Satake M.; Iwase T.; Kurihara M.; Negishi N.; Tada Y.; Yoshida H. Effect of Oxygen Addition to an Argon Plasma on Etching Selectivity of Poly (Methyl Methacrylate) to Polystyrene. J. Micro Nanolithogr., MEMS, MOEMS 2013, 12, 041309 10.1117/1.JMM.12.4.041309.

[ref68] Sarrazin A.; Posseme N.; Pimenta-Barros P.; Barnola S.; Gharbi A.; Argoud M.; Tiron R.; Cardinaud C. PMMA Removal Selectivity to Polystyrene Using Dry Etch Approach. J. Vac. Sci. Technol., B 2016, 34, 061802 10.1116/1.4964881.

[ref69] Reddy A.; Dimitriyev M. S.; Grason G. M. Medial Packing and Elastic Asymmetry Stablize the Double-Gyroid in Block Copolymers. Nat. Commun. 2022, 13, 2629 10.1038/s41467-022-30343-2. 35552400 PMC9098509

[ref70] Reddy A.; Feng X.; Thomas E. L.; Grason G. M. Block Copolymers beneath the Surface: Measuring and Modeling Complex Morphology at the Subdomain Scale. Macromolecules 2021, 54, 9223–9257. 10.1021/acs.macromol.1c00958.

