# Supplementary material for: Epitaxial Growth of Surface Perforations on Parallel Cylinders in Terraced Films of Block Copolymer/Homopolymer Blends
Source: Langmuir. 2024 Mar 29;40(14):7680–91. doi: 10.1021/acs.langmuir.4c00385 (PMC11008238; doi:10.1021/acs.langmuir.4c00385)
Supplement: Supplementary file 1 — la4c00385_si_001.pdf [file la4c00385_si_001.pdf]

Supporting Information

Epitaxial Growth of Surface Perforations on Parallel Cylinders in Terraced Films of Block Copolymer/Homopolymer Blends.

Ya-Sen Sun[1], Yi-Qing Jian[2], Shin-Tung Yang[2], Hsiao-Fang Wang[2], Belda Amelia Junisu[1], Chun-Yu Chen[3], and Jhih-Min Lin[3]*

1. *Department of Chemical Engineering, National Cheng Kung University, Tainan 701, Taiwan*
2. *Department of Chemical and Materials Engineering, National Central University, Taoyuan 32001, Taiwan*
3. *National Synchrotron Radiation Research Center, Hsinchu 30076, Taiwan*

*corresponding author: Y. S. Sun (Email: yssun@gs.ncku.edu.tw)

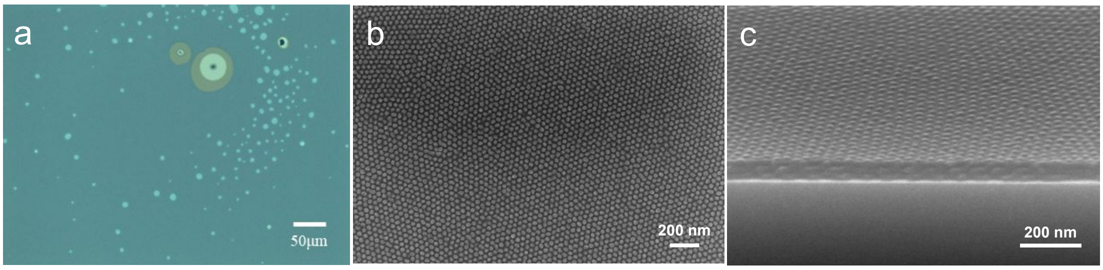

Figure S1. (a) Optical image, (b) top-view and (c) side-view SEM images for a $B_{75}H_{25}$ thin film (h_i~80 nm) that was prolongedly annealed at 230 °C (48h). The SEM images were collected after oxygen plasma etching (15 sec).

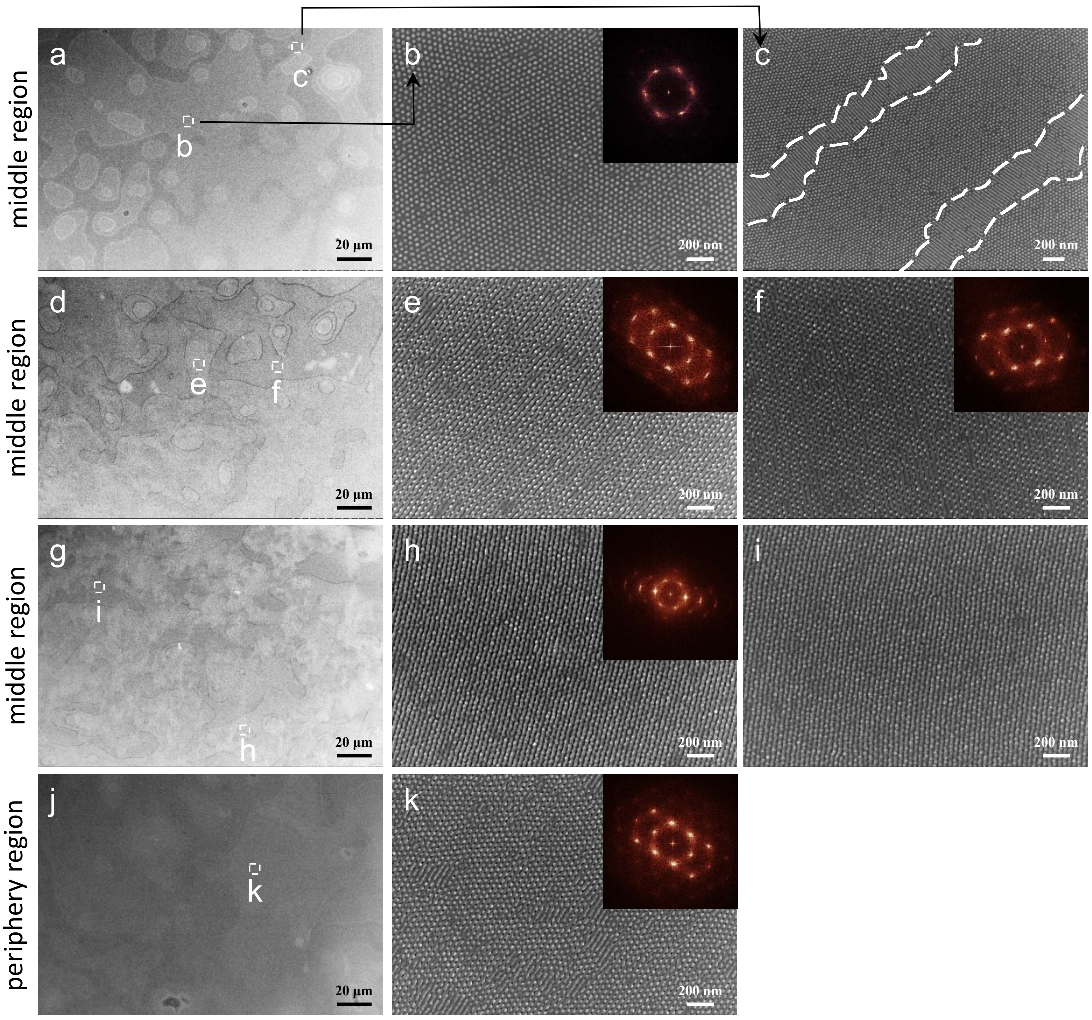

Figure S2. Top-view SEM images for a $B_{75}H_{25}$ film, which was soaked at 310 ^{o}C (10min) and then prolongedly annealed at 230 ^{o}C (48h). The top-view SEM images were recorded at (a–i) three different areas in the center and at (j,k) one area near the periphery. Image c was recorded on the boundaries of relief terraces. The boundaries are highlighted by dot lines in (c). Insets show corresponding FFT patterns.
